# Protein oxidation, nitration and glycation biomarkers for early-stage diagnosis of osteoarthritis of the knee and typing and progression of arthritic disease

**DOI:** 10.1186/s13075-016-1154-3

**Published:** 2016-10-27

**Authors:** Usman Ahmed, Attia Anwar, Richard S. Savage, Paul J. Thornalley, Naila Rabbani

**Affiliations:** 1 Warwick Medical School, Clinical Sciences Research Laboratories, University of Warwick, University Hospital, Coventry, CV2 2DX UK; 2 Warwick Systems Biology Centre, Senate House, University of Warwick, Coventry, CV4 7AL UK

**Keywords:** Osteoarthritis, Rheumatoid arthritis, Machine learning, Oxidative stress, 3-nitrotyrosine, Glycation

## Abstract

**Background:**

There is currently no blood-based test for detection of early-stage osteoarthritis (OA) and the anti-cyclic citrullinated peptide (CCP) antibody test for rheumatoid arthritis (RA) has relatively low sensitivity for early-stage disease. Morbidity in arthritis could be markedly decreased if early-stage arthritis could be routinely detected and classified by clinical chemistry test. We hypothesised that damage to proteins of the joint by oxidation, nitration and glycation, and with signatures released in plasma as oxidized, nitrated and glycated amino acids may facilitate early-stage diagnosis and typing of arthritis.

**Methods:**

Patients with knee joint early-stage and advanced OA and RA or other inflammatory joint disease (non-RA) and healthy subjects with good skeletal health were recruited for the study (n = 225). Plasma/serum and synovial fluid was analysed for oxidized, nitrated and glycated proteins and amino acids by quantitative liquid chromatography-tandem mass spectrometry. Data-driven machine learning methods were employed to explore diagnostic utility of the measurements for detection and classifying early-stage OA and RA, non-RA and good skeletal health with training set and independent test set cohorts.

**Results:**

Glycated, oxidized and nitrated proteins and amino acids were detected in synovial fluid and plasma of arthritic patients with characteristic patterns found in early and advanced OA and RA, and non-RA, with respect to healthy controls. In early-stage disease, two algorithms for consecutive use in diagnosis were developed: (1) disease versus healthy control, and (2) classification as OA, RA and non-RA. The algorithms featured 10 damaged amino acids in plasma, hydroxyproline and anti-CCP antibody status. Sensitivities/specificities were: (1) good skeletal health, 0.92/0.91; (2) early-stage OA, 0.92/0.90; early-stage RA, 0.80/0.78; and non-RA, 0.70/0.65 (training set). These were confirmed in independent test set validation. Damaged amino acids increased further in severe and advanced OA and RA.

**Conclusions:**

Oxidized, nitrated and glycated amino acids combined with hydroxyproline and anti-CCP antibody status provided a plasma-based biochemical test of relatively high sensitivity and specificity for early-stage diagnosis and typing of arthritic disease.

**Electronic supplementary material:**

The online version of this article (doi:10.1186/s13075-016-1154-3) contains supplementary material, which is available to authorized users.

## Background

Oxidation, nitration and glycation of proteins are non-enzymatic processes in tissues and body fluids that produce some of the most quantitatively and functionally important spontaneous post-translational modifications of proteins in clinical disease. Reaction of reactive oxygen species (ROS) with proteins produces a characteristic profile of oxidative damage: trace level oxidized amino acid residues in proteins such as methionine sulfoxide (MetSO), dityrosine (DT), N-formylkynurenine (NFK) and others. Reaction of proteins with reactive nitrogen species (RNS) such as peroxynitrite characteristically forms 3-nitrotyrosine (3-NT). Protein glycation occurs at N-terminal and lysine residue sidechain amino groups to form N_ε_-(1-deoxyfructosyl)lysine (FL) and other fructosamine derivatives, which degrade slowly to form end-stage stable adducts collectively called advanced glycation endproducts (AGEs). Reactive dicarbonyl metabolites such as glyoxal, methylglyoxal (MG) and 3-deoxyglucosone (3-DG) are also important physiological glycating agents and react mainly with arginine residues of proteins to form AGEs. Clinical measurement of protein oxidation, nitration and glycation adducts was recently reviewed [1] (see Additional file 1: Figure S1).

One of the first examples of involvement of oxidative damage in clinical disease was modification of cartilage and other proteins of the joint during development of rheumatoid arthritis (RA) [2, 3]. Increased oxidative damage and nitration to proteins in RA likely arises as a consequence of increased ROS and RNS formed in the respiratory burst of neutrophils and macrophages migrating into the arthritic joint [4]. Osteoarthritis (OA) is also driven by inflammation, at least in the early stages, and may involve increased ROS and RNS exposure [5]. Increased ROS also originates from mitochondrial dysfunction of chondrocytes and other cells implicated in both RA and OA development [6, 7]. Protein glycation has also been implicated in the development of arthritis, which may arise through oxidative stress-driven glycation or “glycoxidation” in RA [8, 9] and through age-related accumulation of AGEs in cartilage, facilitating development of OA [10]. Oxidative damage, nitration and glycation of cartilage is associated with misfolding and aggregation of the proteoglycan–collagen network surrounding chondrocytes and matrix degradation. These damaging modifications are thought to compromise structural integrity and viscoelasticity of cartilage, impairing its ability to sustain mechanical pressures and joint function [11].

In principle, measurement of protein oxidation, nitration and glycation adducts could provide multiple mechanistic biomarkers of arthritic disease to aid in clinical-chemistry-based diagnosis and progression of disease. This is particularly important for detection of early-stage disease. In the detection of early-stage RA (eRA), use of disease-modifying anti-rheumatic drugs (DMARDs) has produced the prospect of a therapeutic cure for RA [12]. Severe life impairment by development of OA may likely be prevented if arthritic disease is identified and treated in the early stages [13].

Currently, magnetic resonance imaging techniques have been developed for evaluation of cartilage damage in early-stage OA (eOA). These imaging techniques have approximately 70 % sensitivity and 90 % specificity compared to reference diagnosis by arthroscopy. They require expensive instrumentation, time and facilities [14]. They cannot be used in patients with implanted pacemakers or aneurysm coils.

Detection of RA by clinical chemistry assessments has been improved by introduction of the anti-cyclic citrullinated peptide (CCP) antibody test, which has 68 % sensitivity and 98 % specificity in established disease [15]. The presumed antigen is citrullinated protein (CP), which we recently identified as increased in both eRA and eOA, with only dominant immunogenicity in the former [16]. The anti-CCP antibody test is now considered state-of-the-art in RA diagnosis. It has relatively low sensitivity for eRA of 61 % and hence, improvement is desirable [15].

In searching for biomarkers for clinical diagnosis, analysis of proteins is often considered and preferred over analysis of amino acids, because proteins are typically longer-lived than amino acids and may be linked functionally to the mechanism of the target disease. Restricted access to samples of affected tissues often precludes measurement of target tissue-based proteins. Proteins damaged by oxidation, nitration and glycation undergo proteolysis to release oxidized, nitrated and glycated amino acids - also called oxidation, nitration and glycation free adducts [1]. Increase in such trace-level damaged amino acids in synovial fluid equilibrates rapidly with plasma. Amino acids may be detected with high analytical specificity and sensitivity and robustly quantified by stable isotopic dilution analysis liquid chromatography-tandem mass spectrometry (LC-MS/MS) [17]. Plasma levels of oxidized, nitrated and glycated amino acids may thereby provide signatures of protein damage, dysfunction and disease progression in the joint in early and advanced stage arthritis. In combination with the biomarker of bone turnover and resorption - hydroxyproline (Hyp) - and anti-CCP antibody status, the signature may be made specific for arthritic disease [15, 16, 18].

In this study we explored changes in the level of oxidized, nitrated and glycated proteins and related trace-level oxidized, nitrated and glycated amino acids in plasma and synovial fluid from subjects with early-stage and advanced or severe OA and RA or non-RA and healthy controls with good skeletal health.

## Methods

### Patients, healthy subjects and sampling

**Fig. 1:**
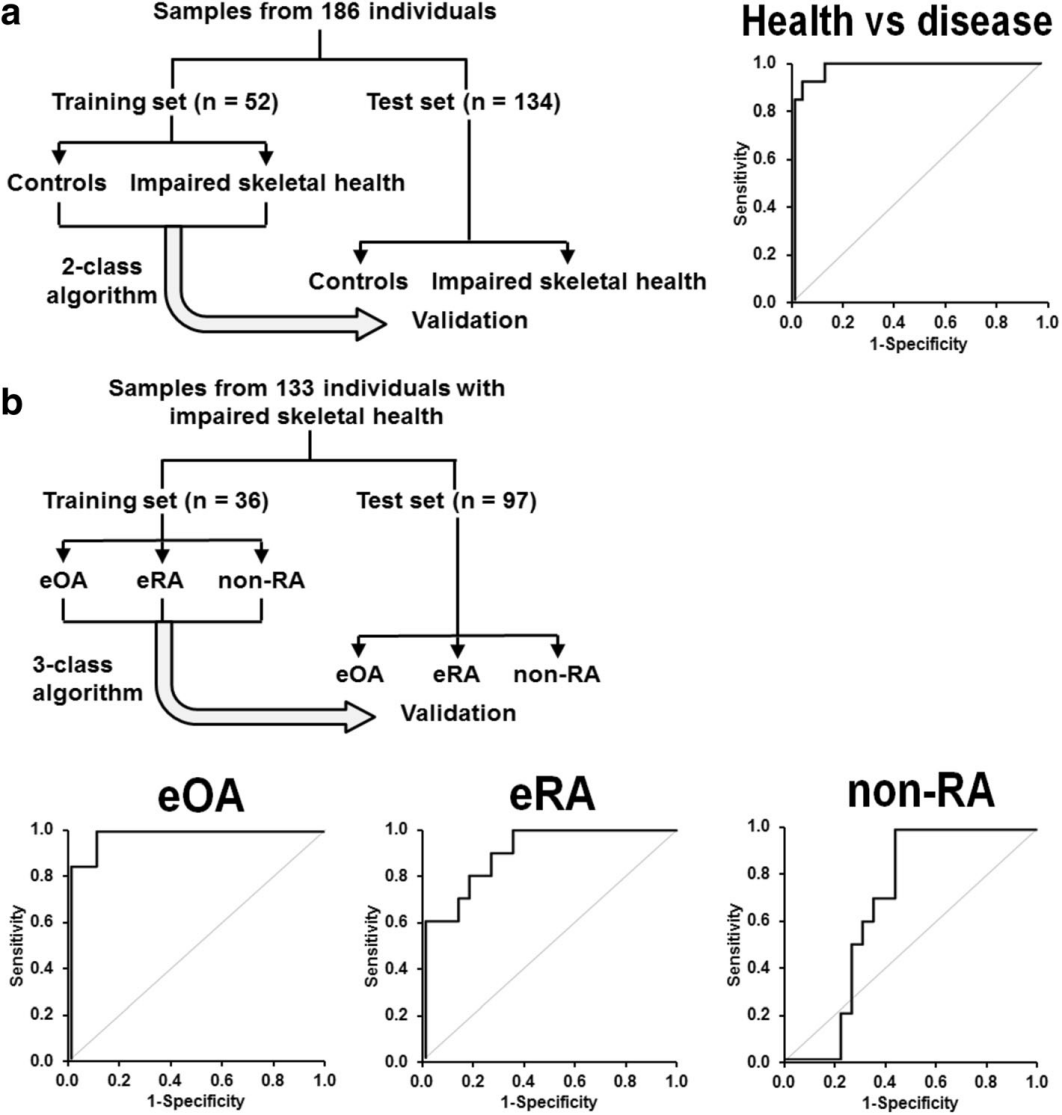
Training and validation of two-stage diagnostic algorithms for detection of impaired skeletal health and discrimination of early-stage osteoarthritis (*eOA*), rheumatoid arthritis (*RA*) and other inflammatory joint disease. **a** Training set and test set study groups for detection of impaired skeletal health. A receiver operating characteristic (ROC) curve is given for the training set. The area under the ROC curve (AUROC) was 0.99 (95 % confidence interval 0.97–1.00). Comparators were eOA + early RA (*eRA*) + non-RA versus healthy controls. A random outcome is 0.50. **b** Training set and test set study groups for discrimination of eOA, eRA and non-RA. ROC curves are given for the training set with AUROC and confidence intervals: eOA, 0.98 (0.96–1.00); eRA, 0.91 (0.81–1.00); and non-RA, 0.68 (0.50–0.86). Comparators were eOA, eRA or non-RA versus other early-stage arthritic diseases combined. A random outcome is 0.33

Patient recruitment, characteristics and sampling was as previously described [16]. The recruited study groups are illustrated in Fig. 1a and b. Briefly, patients with eOA (n = 46), eRA (n = 45) and inflammatory arthritis other than RA (non-RA (n = 42)), and patients with longstanding severe, advanced OA, aOA (n = 17), and advanced RA, aRA (n = 22), were recruited at multiple sites: Orthopaedic Clinics, University Hospital Coventry & Warwickshire (UHCW), Coventry, UK; Department of Rheumatology, Ipswich Hospital NHS Trust, UK; Department of Rheumatology, Royal Devon and Exeter NHS Foundation Trust, Exeter, UK; and Rapid Access Rheumatology Clinic, City Hospital, Birmingham, UK.

Criteria for eOA were: subjects presenting with new-onset knee pain, normal radiographs of the symptomatic knee and routine exploratory arthroscopy with macroscopic findings classified as grade I/II on the Outerbridge scale; recruited at UHCW, Coventry, UK. Patients with eRA and non-RA were recruited within 5 months of the onset of symptoms of inflammatory arthritis at the Rapid Access Rheumatology Clinic, City Hospital, Birmingham, UK. Synovial fluid and peripheral venous blood samples were collected on initial presentation and diagnostic outcomes were determined at follow up. A diagnosis of early rheumatoid arthritis (eRA) was made according to the 1987 American Rheumatoid Association criteria [19]. A diagnosis of non-RA was made where alternative rheumatological diagnoses explained the inflammatory arthritis, as described [20]. For example, training-set non-RA subjects had reactive arthritis (n = 6), pseudogout (n = 1), and unclassified (n = 3). Criteria for aOA were: longstanding or established severe symptoms of OA (≥2 years duration of disease) with corresponding radiographic changes (Kellgren-Lawrence grade IV changes on radiographs) and undergoing therapeutic knee aspiration and corticosteroid instillation or total knee replacement; and recruited at UHCW, Coventry, UK and Ipswich Hospital NHS Trust, Ipswich, UK. Criteria for aRA were: joint stiffness in the mornings of at least one hour duration; symmetrical swelling in three or more joints; radiographic evidence of bone erosions; rheumatoid nodules with increased serum rheumatoid factor (RF); and symptoms of ≥2 years duration [19]. Patients with aRA were recruited at Ipswich Hospital NHS Trust, UK and Royal Devon and Exeter NHS Foundation Trust, Exeter, UK. Criteria for these clinical classifications are similar to those suggested in consensus position and best practice statements [21, 22].

Healthy controls were recruited at participating clinical centres (n = 53). For healthy control subjects the inclusion criteria were no history of joint symptoms, arthritic disease or other morbidity. Exclusion criteria were: history of injury or pain in either knee; taking medication excepting oral contraceptives and vitamins; and any abnormality identified on physical examination of the knee. Control subjects and patients with early-stage disease were recruited as two independent cohorts for a training set and independent test set for data analysis in machine learning methods as explained subsequently.

Peripheral venous blood samples from healthy subjects and patients with eOA were collected after overnight fasting with EDTA anti-coagulant. Synovial fluid from the eOA study group was collected concurrently. Venous blood and synovial fluid samples for eRA, non-RA, aOA and aRA study groups were collected in the non-fasted state. For analytes studied herein, diurnal variation in serum Hyp from healthy subjects was 20 % and for other amino acids up to 13–25 %, depending on the analyte [23, 24]. Diurnal variation in plasma protein glycation and oxidative stress in healthy subjects was <15 %, as judged by plasma fructosamine and plasma cysteine, respectively [25, 26]. Synovial fluid was collected from patients with aOA and aRA and eOA, eRA and with non-RA recruited for the training set cohort. Synovial fluid was not collected from patients with eOA, eRA or non-RA in the test set cohort in which analytes were determined only in plasma/serum.

Blood and synovial fluid were centrifuged (2000 g, 10 minutes) and the plasma and synovial fluid supernatant were removed and stored at –80 °C until analysis. Samples were centrifuged within one hour of collection. A critical time-limiting feature for sample processing is the rate of sample oxidation, as judged by loss of reduced glutathione (GSH) and other thiols, which leads to subsequent increase in protein oxidation adducts (see subsequent text). This prompt sample processing maintains >70 % GSH and thereby avoids artefactual increase in protein oxidation adducts in sample collection [27, 28]. Repeated freeze thawing was avoided as this increases estimates of the protein oxidation adduct, methionine sulfoxide [29].

Serum was available for the eRA and non-RA study groups and plasma was available for all others. Serum is comparable to plasma as a sample matrix where the major protein lost during clotting (fibrinogen) does not affect median glycation, oxidation and nitration adduct residue content normalised to amino-acid-modified and related free adduct and Hyp concentrations [1, 16].

### Analysis of oxidized, nitrated and glycated protein and oxidation, nitration and glycation free adducts in plasma/serum and synovial fluid

The contents of oxidation, nitration and glycation adduct residues in plasma/serum and synovial proteins were quantified in exhaustive enzymatic digests by stable isotopic dilution analysis LC-MS/MS, with correction for autohydrolysis of hydrolytic enzymes as described [17]. Oxidation, nitration and glycation free adducts were determined in the ultrafiltrates of the same samples. Ultrafiltrate of plasma/serum or synovial fluid (100 μl) was collected by microspin ultrafiltration (10 kDa cutoff) at 4 °C. Retained protein was diluted with water to 500 μl and washed by four cycles of concentration to 50 μl and dilution to 500 μl with water over the microspin ultrafilter at 4 °C. The final washed protein (100 μl) was delipidated and hydrolysed enzymatically as described [16, 17].

Protein hydrolysate (25 μl, 32 μg protein equivalent) or ultrafiltrate was mixed with stable isotopic standard analytes (for amounts see Additional file 1: Table S1) and analysed by LC-MS/MS using an Acquity™ UPLC system with a Quattro Premier tandem mass spectrometer (Waters, Manchester, U.K.) [17]. Samples are maintained at 4 °C in the autosampler during batch analysis. The columns were: 2.1 × 50 mm and 2.1 mm × 250 mm, 5 μm particle size Hypercarb™ (Thermo Scientific), in series with programmed switching, at 30 °C. Chromatographic retention is necessary to resolve oxidized analytes from their amino acid precursors to avoid interference from partial oxidation of the latter in the electrospray ionization source of the mass spectrometric detector. Analytes were detected by electrospray positive ionization and mass spectrometry multiple reaction monitoring (MRM) mode where analyte detection response is specific for mass/charge ratio of the analyte molecular ion and major fragment ion generated by collision-induced dissociation in the mass spectrometer collision cell. The ionization source and desolvation gas temperatures were 120 °C and 350 °C, respectively, cone gas and desolvation gas flow rates were 99 and 900 l/h and the capillary voltage was 0.60 kV. Argon gas (5.0 × 10^-3^ mbar) was in the collision cell.

For MRM detection, molecular ion and fragment ion masses and collision energies optimized to ± 0.1 Da and ± 1 eV, respectively, were programmed (Additional file 1: Table S1). Analytes determined were: oxidation adducts - MetSO, DT and NFK; nitration adduct –3-NT; and glycation adducts - FL, and advanced glycation endproducts (AGEs), N_ε_-carboxymethyl-lysine (CML), N_ε_-(1-carboxyethyl)lysine (CEL), N_ω_-carboxymethylarginine (CMA), hydroimidazolones derived from glyoxal, methylglyoxal and 3-deoxyglucosone (G-H1, MG-H1 and 3DG-H), respectively), pentosidine and methylglyoxal-derived lysine dimer (MOLD); and others - Hyp and amino acids - arg, lys, tyr, trp, met and val (Additional file 1: Figure S1). Valine is determined in protein hydrolysates for the protease autohydrolysis correction [17]. Oxidation, nitration and glycation adduct residues are normalised to their amino acid residue precursors and given as mmol/mol amino acid modified; and related free adducts are given in nM. Chemical structures and biochemical and clinical significance of these analytes have been described elsewhere [1, 16].

### Other assessments

Anti-CCP antibody positivity was assessed by automated enzymatic immunoassay (EliA CCP; Phadia, Uppsala, Sweden).

### Machine learning analysis

The objective was to distinguish between the following four groups: healthy control, eOA, eRA and non-RA. In all cases, the diagnostic algorithms were trained on the training data set, before being used to predict the disease class for each sample in the test data set (Fig. 1a and b). A two-stage approach was taken: (1) to distinguish between disease and healthy control; and (2) to distinguish between eOA, eRA and non-RA. The outcome was to assign, for each test set sample, a set of probabilities corresponding to each of the disease/control groups - the group assignment being that for which the probability is highest. Test data were held separate from algorithm training; algorithm settings were not adjusted once we began to analyse the test set data, thereby guarding against overfitting and hence providing a rigorous estimate of predictive performance. We also performed fivefold cross-validation separately on each of the training, test data sets, to assess predictive performance internally for each data set. Four algorithm types were tested for performance using random forests, multi-class logistic regression, multi-class sparse logistic regression, and support vector machines [30–32]. In the training set cross-validations, we use a panel of 15 plasma biomarkers: RF, anti-CCP antibody positivity, Hyp and MetSO, DT, NFK, 3-NT, FL, CML, CEL, CMA, G-H1, MG-H1, 3DG-H and pentosidine free adducts. Methylglyoxal-derived lysine dimer (MOLD) was omitted as levels were close to the limit of detection.

We used the area under the curve of the receiver operating characteristic (ROC) plot (AUROC) statistic as our measure of performance [33], with the 95 % CI determined via bootstrap analysis using the R package pROC [34]. In the training-set test set cross-validation we used the minimum set of features giving the maximum AUROC. This minimum feature set was used in the test set validation. We produced sensitivity/specificity values from the ROC curves using an automated procedure that finds where the sensitivity/specificity values are most similar. Where we consider three classes, we consider a set of one-versus-all ROC curves (one per class). Fivefold cross-validation was carried out on the training set data to give an initial estimate of predictive performance, and to identify the best-performing machine learning method. We then trained the algorithm on the entire training set, before making predictions for the test data set. We also performed fivefold cross-validation on the test set data. Data were analysed using R version 3.1.3.

### Statistical analyses

Non-normally distributed variables are summarized as median (lower to upper quartile), two groups were compared using the Mann–Whitney *U* test and four groups were compared using the Kruskal-Wallis test for independent samples and Wilcoxon’s signed ranks test for two-group, paired samples (plasma and synovial fluid from the same donor). Correlation analysis was performed using the Spearman method. Data were analysed using SPSS, version 22.0. A Bonferroni correction of 13 was applied for testing of multiple protein oxidation, nitration and glycation adduct levels, assuming a null hypothesis that any of the 13 adducts measured may have levels that are significantly changed in the arthritic disease study groups. Data were considered significantly different when the *P* value was <0.05/13 or <0.0038. This report adheres to the guidelines for transparent reporting of a multivariable prediction model for individual prognosis or diagnosis (TRIPOD statement) [35].

## Results

### Summary description of protein oxidation, nitration and glycation adduct changes in the plasma and synovial fluid and inter-compartment correlations in patients with early and advanced arthritis

**Fig. 2:**
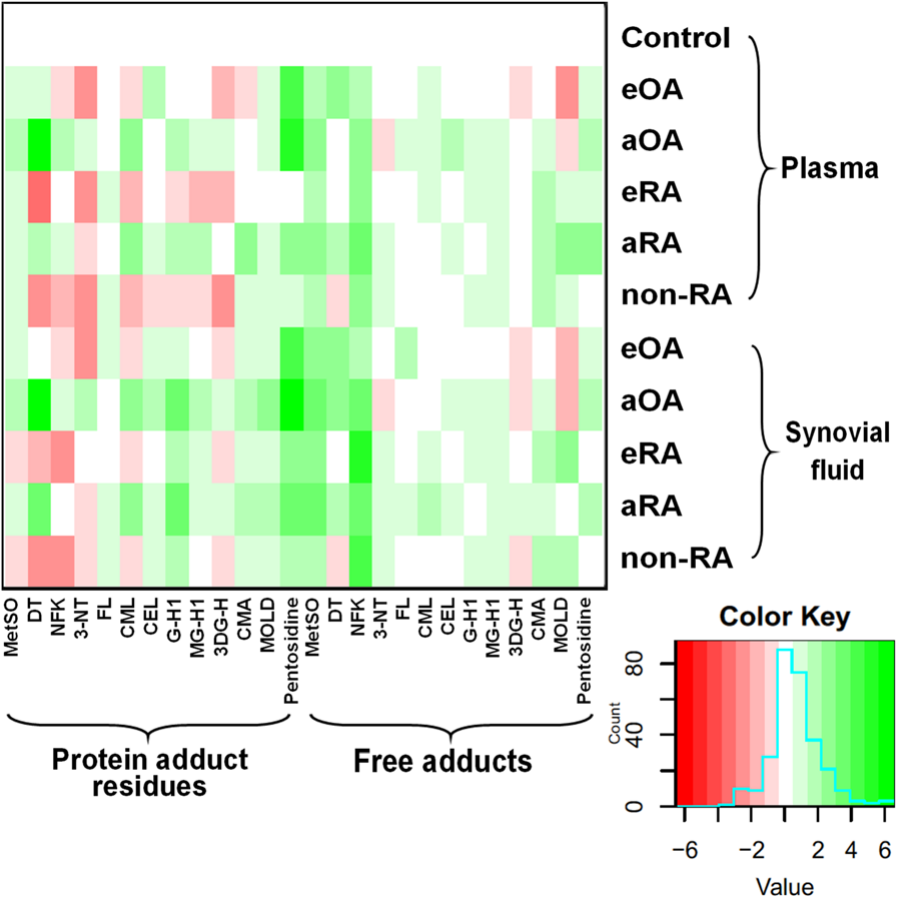
Heat map of changes in glycated, oxidized and nitrated proteins and amino acids in plasma and synovial fluid from patients with early and advanced arthritis. Data are given in Additional file 1: Tables S2–S7. Heat map values are on a log_2_ scale, normalised to levels in plasma from healthy control subjects, with the key and frequency of changes (*light blue line*) given (*inset*). *eOA* early osteoarthritis, *aOA* advanced osteoarthritis, *eRA* early rheumatoid arthritis, *aRA* advanced rheumatoid arthritis *CML* N_ε_-carboxymethyl-lysine, *CEL* N_ε_-(1- carboxyethyl) -lysine, *MetSO* methionine sulfoxide, *DT* dityrosine, *FL* N_ε_-fructosyl-lysine, *MG-H1* methylglyoxal-derived hydroimidazolone, *3DG-H* 3-deoxyglucosone-derived hydroimidazolone isomers, *NFK* N-formylkynurenine, *3-NT* 3-nitrotyrosine, *G-H1* glyoxal-derived hydroimidazolone, *CMA* N_ω_-carboxymethylarginine, *MOLD* methylglyoxal-derived lysine dimer

Changes in protein oxidation, nitration and glycation status in plasma and synovial fluid in patients with eOA, aOA, eRA, aRA and non-RA with respect to plasma levels in healthy controls are summarised in a heat map (Fig. 2). Data and detailed descriptions of the changes are given in Additional file 1: Supplementary information and Tables S2–S7, including a description of the effects of drug therapy in aRA. The heat map indicates there were changes in protein oxidation, nitration and glycation adduct levels in both adduct contents of protein and free adduct concentrations in plasma and synovial fluid. The different patterns of adduct levels provides the basis to detect and distinguish the presence and type of arthritis at the early stage of clinical development, and in advanced-stage disease.

In plasma for healthy controls and early-stage disease (eOA, eRA and non-RA), there were changes in oxidation, nitration and glycation adduct contents of protein (MetSO, DT, 3-NT, FL, MG-H1, CMA and pentosidine) and oxidation and glycation free adduct concentrations (MetSO, NFK, DT, CMA and MOLD; *P* < 0.05, Kruskal-Wallis), indicating disrupted proteostasis in protein glycation, oxidation and nitration in the plasma compartment and tissue release into plasma of free adducts from proteolysis of oxidized and glycated proteins. Similar analysis for early-stage and advanced disease of the same type showed the following changes in plasma: for aOA versus eOA, increased NFK, DT, 3-NT, CML and 3DG-H adducts of protein and increased MetSO, NFK, DT, 3-NT, CEL and CMA free adducts; and for aRA versus eRA, increased DT, 3-NT, FL, CML, G-H1, MG-H1 and CMA adducts of protein and increased MetSO, DT and pentosidine free adducts.

To assess if changes of analyte in the synovial fluid are reflected by similar increases in plasma we computed inter-compartment homotypic correlation (correlation between levels of a marker in plasma and synovial fluid from the same subject) (Table 1). For eOA and eRA combined, in early-stage disease there were 10 homotypic correlations between synovial fluid and plasma for oxidized, nitrated and glycated amino acids and only 5 homotypic correlations for oxidation nitration and glycation adducts of protein. For aOA and aRA combined, in advanced disease there were 9 homotypic correlations between synovial fluid and plasma for oxidized, nitrated and glycated amino acids and 13 homotypic correlations for oxidation nitration and glycation adducts of protein: in non-RA there were 3 homotypic correlations for amino acids (3 glycated amino acids) and one homotypic correlation for protein (glycated protein). This suggests that oxidized, nitrated and glycated amino acids equilibrate readily between synovial fluid and plasma compartments in early-stage arthritis and both oxidized, nitrated and glycated amino acids and proteins equilibrate between synovial fluid and plasma compartments in advanced-stage arthritis.

**Table 1:** Homotypic correlation of protein oxidation, nitration and glycation adducts in synovial fluid and plasma compartments

Study group	Protein adduct residue	Free adduct
eOA	CML (*r* = 0.60, *P* < 0.05), CEL (*r* = 0.82, *P* < 0.001), MetSO (*r* = 0.61, *P* < 0.05), DT (*r* = 0.53, *P* < 0.05)	CEL (*r* = 0.59, *P* < 0.05), 3DG-H (*r* = 0.62, *P* < 0.05), pentosidine (*r* r = 0.71)
aOA	FL (*r* = 0.58, *P* < 0.05), MG-H1 (*r* = 0.80, *P* < 0.001)	FL (*r* = 0.75, *P* < 0.01), CEL (*r* = 0.63, *P* < 0.05), G-H1 (*r* = 0.71, *P* < 0.01), MG-H1 (r = 0.91, P < 0.001), NFK (r = 0.73, P < 0.05)
	3DG-H (*r* = 0.72, *P* < 0.01), MetSO (*r* = 0.63, *P* < 0.05)	
eRA	Pentosidine (*r* = 0.82, *P* < 0.01)	FL (*r* = 0.87, *P* < 0.01), CML (*r* = 0.67, *P* < 0.05), CEL (*r* = 0.81, *P* < 0.01), MG-H1 (*r* = 0.92, *P* < 0.001), 3DG-H (*r* = 0.83, *P* < 0.01), CMA (0.72, *P* < 0.05), pentosidine (*r* = 0.78, *P* < 0.01)
aRA	CML (*r* = 0.56, *P* < 0.05), CEL (*r* = 0.70, *P* < 0.01), G-H1 (*r* = 0.52, *P* < 0.05), MG-H1 (*r* = 0.86, *P* < 0.001), 3DG-H (*r* = 0.86, *P* < 0.001), CMA (0.50, *P* < 0.05), pentosidine (*r* = 0.83, *P* < 0.01), NFK (*r* = 0.56, *P* < 0.05), DT (*r* = 0.64, *P* < 0.01)	G-H1 (*r* = 0.53, *P* < 0.05), MG-H1 (*r* = 0.87, *P* < 0.001)
		3DG-H (*r* = 0.73, *P* < 0.01), 3-NT (0.67, *P* < 0.01)
Non-RA	Pentosidine (*r* = 0.76, *P* < 0.05)	CEL (*r* = 0.73, *P* < 0.05), MG-H1 (*r* = 0.76, *P* < 0.05), MOLD (*r* = 0.81, *P* < 0.01)

*eOA* early osteoarthritis, *aOA* advanced osteoarthritis, *eRA* early rheumatoid arthritis, *aRA* advanced rheumatoid arthritis, *CML* N_ε_-carboxymethyl-lysine, *CEL* N_ε_-(1- carboxyethyl)-lysine, *MetSO* methionine sulfoxide, *DT* dityrosine, *FL* N_ε_-fructosyl-lysine, *MG-H1* methylglyoxal-derived hydroimidazolone, *3DG-H* 3-deoxyglucosone-derived hydroimidazolone isomers, *NFK* N-formylkynurenine, *3-NT* 3-nitrotyrosine, *G-H1* glyoxal-derived hydroimidazolone, *CMA* N_ω_-carboxymethylarginine

### Machine learning analysis

To explore the diagnostic utility of protein damage measurements we analysed plasma amino acid analyte data by a machine learning approach. Random forests was the best-performing method. Application of two algorithms consecutively gave the best diagnostic outcome. First, with algorithm 1 we discriminated between healthy control and early-stage arthritis. Second, with algorithm 2 we identified types of arthritis - eOA, eRA and non-RA. Algorithms were trained with a training set subject group and validated independently in a different test set subject group (Fig. 1). Training set data are in Additional file 1: Tables S3, S6 and S7 and test set data are in Additional file 1: Tables S8–S10. Algorithm features for maximum AUROC were:

1. Disease (eOA, eRA and non-RA) versus healthy control: Hyp, MetSO, DT, NFK, 3-NT, CEL, CMA, G-H1, MG-H1, 3DG-H and pentosidine free adducts. The AUROC value was 0.99, 0.96 and 0.77, for the training set and test set cross-validations and test set validation, respectively. A random outcome is 0.50. Related sensitivity/specificity was 0.92/0.91, 0.89/0.90 and 0.73/0.72, respectively (Fig. 1a, Table 2 (algorithm feature) and Table 3 (confusion matrices)).

**Table 2:** Predictive algorithm outcomes using the random forest algorithm diagnostic characteristics

	Algorithm 1	Algorithm 2		
Features	Hyp and MetSO, DT, NFK, 3-NT, CEL, CMA, G-H1, MG-H1, 3DG-H and pentosidine	anti-CCP antibody positivity and MetSO, DT, 3-NT, FL, CML, CEL, CMA, MG-H1, 3DG-H and pentosidine		
Comparators	Disease versus control	eOA	eRA	non-RA
Training set cross-validation				
Correct, *n*	44/46	13/13	6/8	8/12
Sensitivity	0.92 (0.64–1.00)	0.92 (0.64–1.00)	0.80 (0.44 - 0.97)	0.70 (0.35 - 0.93)
Specificity	0.91 (0.76–0.98)	0.90 (0.68–0.99)	0.78 (0.56–0.93)	0.65 (0.43–0.84)
AUROC	0.99 (0.97–1.00)	0.98 (0.96–1.00)	0.91 (0.82–1.00)	0.68 (0.49–0.86)
Positive predictive value	1.0	1.0	0.60	0.80
Negative predictive value	0.85	1.0	0.84	0.83
Positive likelihood ratio	10.2	9.2	3.6	2.0
Negative likelihood ratio	0.09	0.09	0.26	0.46
Test set cross-validation				
Correct, *n*	119/134	30/31	22/27	27/39
Sensitivity	0.89 (0.75–0.97)	0.83 (0.65–0.94)	0.77 (0.6–0.9)	0.72 (0.53–0.86)
Specificity	0.90 (0.82–0.95)	0.84 (0.73–0.92)	0.76 (0.63–0.86)	0.71 (0.58–0.81)
AUROC	0.96 (0.93–0.99)	0.93 (0.88–0.98)	0.87 (0.8–0.94)	0.77 (0.68–0.86)
Positive predictive value	0.96	0.97	0.81	0.69
Negative predictive value	0.75	1.0	0.81	0.91
Positive likelihood ratio	8.9	5.2	3.2	2.5
Negative likelihood ratio	0.12	0.20	0.30	0.39
Test set validation				
Correct, *n*	63/134	18/19	8/35	31/32
Sensitivity	0.73 (0.56–0.86)	0.83 (0.65–0.94)	0.60 (0.42–0.76)	0.81 (0.64–0.93)
Specificity	0.72 (0.62–0.81)	0.84 (0.73–0.92)	0.61 (0.46–0.72)	0.80 (0.68–0.89)
AUROC	0.77 (0.69–0.85)	0.91 (0.84–0.99)	0.62 (0.5–0.75)	0.84 (0.77–0.92)
Positive predictive value	0.62	0.60	0.23	0.97
Negative predictive value	0.05	0.99	0.97	0.43
Positive likelihood ratio	2.6	5.2	1.5	4.1
Negative likelihood ratio	0.38	0.20	0.67	0.24

The 95 % CI for sensitivity and specificity are given in brackets. *eOA* early osteoarthritis, *aOA* advanced osteoarthritis, *eRA* early rheumatoid arthritis, *aRA* advanced rheumatoid arthritis, *CML* N_ε_-carboxymethyl-lysine, *CEL* N_ε_-carboxyethyl-lysine, *MetSO* methionine sulfoxide, *DT* dityrosine, *FL* Nε-fructosyl-lysine, *MG-H1* methylglyoxal-derived hydroimidazolone, *3DG-H* 3-deoxyglucosone-derived hydroimidazolone isomers, *NFK* N-formylkynurenine, *3-NT* 3-nitrotyrosine, *G-H1* glyoxal-derived hydroimidazolone, *CMA*N_ω_-carboxymethylarginine

**Table 3:** Predictive algorithm outcomes using the random forest: confusion matrices

	Algorithm 1			Algorithm 2		
Clinical class	Predicted class		Clinical class	Predicted class		
	Control	Disease		eOA	eRA	Non-RA
Training set cross-validation						
Control	11	2	eOA	13	0	0
Disease	0	33	eRA	0	6	4
			Non-RA	0	2	8
Test set cross-validation						
Control	33	4	eOA	30	0	0
Disease	11	86	eRA	1	22	12
			Non-RA	0	5	27
Test set validation						
Control	2	35	eOA	18	2	10
Disease	36	61	eRA	0	8	27
			Non-RA	1	0	31

*eOA* early osteoarthritis, *aOA* advanced osteoarthritis, *eRA* early rheumatoid arthritis, *aRA* advanced rheumatoid arthritis, *CML* N_ε_-carboxymethyl-lysine, *CEL* N_ε_-(1- carboxyethyl)-lysine, *MetSO* methionine sulfoxide, *DT* dityrosine, *FL* Nε-fructosyl-lysine, *MG-H1* methylglyoxal-derived hydroimidazolone, *3DG-H* 3-deoxyglucosone-derived hydroimidazolone isomers, *NFK* N-formylkynurenine, *3-NT* 3-nitrotyrosine, *G-H1* glyoxal-derived hydroimidazolone, *CMA* N_ω_-carboxymethylarginine
2. Typing of eOA, eRA and non-RA: anti-CCP antibody positivity, MetSO, DT, 3-NT, FL, CML, CEL, CMA, MG-H1, 3DG-H and pentosidine free adducts.

For eOA, eRA and non-RA, AUROC values were in the range 0.68–0.98, 0.77–0.93 and 0.62–0.91 for training set and test set cross-validations and test set validation, respectively. A random outcome is 0.33 (three disease classes). Omission of anti-CCP antibody positivity assessment decreased the AUROC by 0.0023. Related sensitivities/specificities for eOA, eRA and non-RA were: 0.92/0.90, 0.80/0.78 and 0.70/0.65; 0.83/0.84, 0.77/0.76 and 0.72/0.71; and 0.83/0.84, 0.60/0.61 and 0.81/0.80 (Fig. 1b and Table 2 (algorithm feature) and Table 3 (confusion matrices)).

## Discussion

Increased protein oxidation, nitration and glycation of cartilage have long been implicated as damaging modifications in the joint, mediating development of arthritic disease [2, 3, 8]. There have been few studies on protein damage in early-stage disease. Experimental studies of gene deletion and pharmacological activation of nuclear factor erythroid 2-related factor 2 (Nrf2), a transcription factor that coordinates basal and inducible expression of enzymes protective against oxidative stress and dicarbonyl glycation [36, 37], supports a role of oxidative damage and dicarbonyl glycation in the development of OA and RA [38, 39]. Trace-level oxidation, nitration and glycation free adducts potentially indicate the presence of this in plasma.

Early-stage detection of arthritic disease and effective lifestyle and/or pharmaceutical interventions could markedly decrease morbidity in OA, achieve a cure for RA and improve outcomes for other arthritic disease [12, 13]. Development of drugs to suppress oxidative damage and glycation damage to collagen in arthritis will be facilitated by availability of companion diagnostic tests based on related biomarkers [40]. There is no established method to detect eOA. Radiography remains the method of choice for later staging of aOA but shows little or no change in eOA. The anti-CCP antibody test is now a clinical standard biochemical test for RA and diagnosis is often confirmed before damage to the joint occurs. Even for eRA, however, the anti-CPP antibody test has relatively low sensitivity [15]; indeed, we observed sensitivity of 56 % herein with patients recruited within 5 months of the onset of symptoms [16]. Hitherto, biomarkers of protein oxidation, nitration and glycation have not been developed for clinical diagnostic application in arthritis, excepting studies on plasma 3-NT [41]; the trace-level of these biomarkers leads to them often being overlooked in untargeted approaches to biomarkers in arthritis [42].

Herein we comprehensively and quantitatively measured protein glycation, oxidation and nitration adducts in plasma and synovial fluid in arthritis. Analysis for diagnostic application suggests a rapid plasma-based test derived for diagnosis of presence and typing of early-stage arthritis suitable for skeletal health screening based on plasma oxidized, nitrated and glycated amino acids in combination with Hyp and the anti-CCP antibody test is available and suitable for further validation and development.

In developing an improved test for detection and typing of early-stage arthritis we focussed on the trace glycated, oxidized and nitrated amino acids in plasma as reporters of damaging modifications of proteins in joints. Plasma is readily accessed, ultrafiltrate easily prepared and analytes rapidly and robustly quantifiable by stable isotopic dilution analysis LC-MS/MS [1]. This technology is available in most clinical chemistry laboratories; it is the most stable mass spectrometry platform and relatively inexpensive to implement, with analyte interference-free multiplexing and low cost. A rapid escape rate of amino acids in synovial fluid to plasma [43, 44] suggested that plasma/serum is a suitable surrogate sample for synovial fluid. The findings of similar levels of analytes in synovial fluid and plasma in patients and positive correlation between plasma and synovial fluid levels of analytes for the different study groups support this.

Basing our diagnostic algorithm test on oxidized, nitrated and glycated amino acids rather than oxidized, nitrated and glycated proteins provided for simple and rapid pre-analytic processing; *cf.* 4 days for enzymatic hydrolysis of proteins [17]. Changes in normal, undamaged amino acids of plasma in advanced arthritis were studied previously but had limited diagnostic utility [45, 46]; the damaging modifications of oxidation, nitration and glycation provide a mechanistic link to arthritic disease and thereby diagnostic significance. Oxidized, nitrated and glycated amino acids are changed in other disease conditions [47–49]; combination of the responses with Hyp, however, make for specific detection of arthritic disease [16].

Surprisingly, there was evidence of decreased oxidation, nitration and glycation adducts in proteins of early-stage disease in eOA, eRA and non-RA. This may relate to increased capillary permeability and increased transcapillary escape rate (TER) of albumin in early-stage disease [50]. Albumin thereby has shorter residence time in plasma and longer residence time in interstitial fluid in the peripheral tissues at a lower concentration and likely in the presence of lower concentrations of oxidizing, nitrating and glycating agents. It will thereby have an overall decreased rate of oxidation, nitration and glycation. Increased capillary permeability has been observed in RA [51]. This effect is lost when marked increase in protein glycation, oxidation and nitration in the joints in advanced disease outweighs the effects of increased protein residence time at interstitial sites. Albumin TER has been described previously as a confounding factor in the diagnostic use of plasma protein glycation [52].

In the two-step algorithm-based diagnosis, feature selection by the data-driven machine learning process produced algorithms with multiple oxidation, nitration and glycation adducts, suggesting estimates of these adducts contribute to the diagnostic power to discriminate both disease versus control and also typing of early-stage disease. Hyp was important in discriminating disease versus control but not for typing of early-stage arthritis. This is likely because increased Hyp was common to all types of early-stage arthritis [16]. Similarly, anti-CCP antibody status was important in discriminating types of arthritis and hence was a feature only in the second algorithm. This analysis builds on the wealth of experience and diagnostic value of anti-CCP antibody positivity in the detection of RA [15].

AUROC values were very high (0.99 for skeletal health versus disease, and 0.98 for eOA), indicating a high probability of correctly identifying early-stage OA. Omission of the anti-CCP antibody positivity assessment gave only a minor improvement in AUROC for arthritis typing and so may be omitted with minor loss of performance. These were observed particularly for cross-validations in both training set and test set data and were lower in independent test set validation. Further validation is required in independent patient cohorts and study of the association of the diagnostic response to clinical symptoms and radiographic changes. There is also potential for monitoring of disease progression.

There have been no similar machine learning studies excepting our previous study with plasma Hyp and CP [16]. AUROC values herein were markedly improved for detection of healthy controls, eOA and non-RA, and slightly decreased for eRA (0.93 versus 0.98). Addition of plasma CP estimates from our previous study [16] in the algorithm optimisation herein did not improve diagnostic performance. Other studies have developed algorithms based on measurement of cytokines but for advanced OA and RA [53]. Clinical detection of early-stage arthritis herein is potentially advantageous. Other serum markers for eOA detection have been suggested - disintegrin and metalloproteinase with thrombospondin motifs (ADAMTS)-4, ADAMTS-5, matrix metalloproteinase (MMP)-1 and MMP-3 - but sensitivity and specificity was not reported [54]. The diagnostic performance of the two-step algorithm herein improves significantly on other current candidate biomarkers proposed for early-stage arthritis [16, 55, 56].

## Conclusions

There were characteristic profiles of protein oxidation, nitration and glycation adducts in protein from synovial fluid and plasma, and related oxidized, nitrated and glycated amino acids, in good skeletal health and arthritis disease, with changes in the latter associated with classification and severity of disease. Combination of estimates of oxidized, nitrated and glycated amino acids with hydroxyproline and anti-CCP antibody status in plasma provided a biochemical test of relatively high sensitivity and specificity for early-stage diagnosis and typing of arthritic disease.

## Additional file

Additional file 1: Supplementary information. Detailed description of changes in protein oxidation, nitration and glycation in plasma and synovial fluid from patients with early and advanced arthritis, including effect of drug therapy on protein oxidation, nitration and glycation adducts in plasma and synovial fluid of patients with advanced rheumatoid arthritis and supplementary Tables S1–S10. Figure S1 shows the molecular structures of the amino acid analytes determined. (DOCX 841 kb)

## Abbreviations

3-DG: 3-deoxyglucosone
3DG-H: 3-deoxyglucosone-derived hydroimidazolone isomers
3-NT: 3-nitrotyrosine
ADAMTS: a disintegrin and metalloproteinase with thrombospondin motifs
AGE: advanced glycation endproduct
aOA: advanced osteoarthritis
aRA: advanced rheumatoid arthritis
AUROC: area under the curve of the receiver operating characteristic plot
CCP: cyclic citrullinated peptide
CEL: N_ε_-(1-carboxyethyl)lysine
CMA: N_ω_-carboxymethylarginine
CML: N_ε_-carboxymethyl-lysine
CP: citrullinated proteins
DMARD: disease-modifying anti-rheumatic drug
DT: dityrosine
EDTA: ethylenediaminetetra-acetic acid
eOA: early-stage osteoarthritis
eRA: early-stage rheumatoid arthritis
FL: N_ε_-fructosyl-lysine
G-H1: glyoxal-derived hydroimidazolone
Hyp: hydroxyproline
LC-MS/MS: liquid chromatography-tandem mass spectrometry
MetSO: methionine sulfoxide
MG: methylglyoxal
MG-H1: methylglyoxal-derived hydroimidazolone
MMP: matrix metalloproteinase
MOLD: methylglyoxal-derived lysine dimer
NFK: N-formylkynurenine
non-RA: inflammatory joint disease other than rheumatoid arthritis (often self-resolving)
OA: osteoarthritis
RA: rheumatoid arthritis
RF: rheumatoid factor
RNS: reactive nitrogen species
ROC: receiver operating characteristic
ROS: reactive oxygen species
TER: transcapillary escape rate
TROPID: transparent reporting of a multivariable prediction model for individual prognosis or diagnosis
UPLC: ultra high performance liquid chromatography
