# Supplementary material for: Protein oxidation, nitration and glycation biomarkers for early-stage diagnosis of osteoarthritis of the knee and typing and progression of arthritic disease
Source: Arthritis Res Ther. 2016 Oct 27;18:250. doi: 10.1186/s13075-016-1154-3 (PMC5081671; doi:10.1186/s13075-016-1154-3)
Supplement: Additional file 1: — Supplementary information. Detailed description of changes in protein oxidation, nitration and glycation in plasma and synovial fluid from patients with early and advanced arthritis, including effect of drug therapy on protein oxidation, nitration and glycation adducts in plasma and synovial fluid of patients with advanced rheumatoid arthritis and supplementary Tables S1–S10. Figure S1 shows the molecular structures of the amino acid analytes determined. (DOCX 841 kb) [file 13075_2016_1154_MOESM1_ESM.docx]

Supplementary information

**Protein oxidation, nitration and glycation biomarkers for early-stage diagnosis, typing and progression of arthritic disease**

Usman Ahmed *et al.*

**Detailed description of changes in protein oxidation, nitration and glycation in plasma and synovial fluid of patients with early and advanced arthritis.**

#### Oxidation adducts of protein in plasma and synovial fluid

MetSO residue content of plasma protein was increased *ca.* 2-fold in patients with eOA, eRA, aRA and non-RA and increased *ca.* 3-fold in patients with aOA, with respect to healthy controls. MetSO residue content of synovial fluid protein was increased 2 – 3 fold in eOA and aOA whereas it was decreased 53% in eRA, with respect to plasma protein content of healthy controls. MetSO residue content of synovial fluid protein of patients with aRA was increased *ca.* 3-fold with respect to patients with eRA. NFK residue content of plasma protein was little changed in the study groups except for *ca.* 3-fold increase in patients with aOA with respect to patient with eOA. The NFK residue content of synovial fluid protein was decreased 85% and 83% in patients with non-RA and eRA, with respect to NFK residue content of plasma protein of healthy controls. There was also a *ca.* 5-fold increase in NFK residue content of synovial fluid protein in aOA, with respect to NFK residue content of plasma protein of healthy controls. DT residue content of plasma protein was increased *ca.* 55-fold and 56-fold in patients with aRA and aOA whereas it was decreased 68% and 74% in patients with eRA and non-RA, respectively, with respect to healthy controls. DT residue content of plasma protein was markedly increased in advanced versus early-stage disease: DT residue content of plasma protein was increased *ca.* 30 fold in aOA with respect to eOA and increased *ca.* 14-fold in aRA with respect eRA. Similar effects were found for DT residue content of synovial fluid protein: DT residue content of synovial fluid protein was increased *ca.* 88 fold in aOA with respect to eOA and increased *ca.* 29-fold in aRA with respect to eRA. Surprisingly, 3-NT residue content of plasma protein decreased in patients with eOA (-85%), eRA (- 83%) and non-RA (- 80%) but remained unchanged in patients with aOA and aRA, with respect to healthy controls. In synovial fluid, the 3-NT residue content of protein was decreased 83% in patients with eOA and increased *ca.* 4-fold in patients with aOA. 3-NT residue content was increased *ca.* 6-fold in synovial fluid protein, compared to plasma protein, of patients with aOA and eRA (Table S2).

#### Oxidation and nitration free adducts of plasma and synovial fluid

MetSO free adduct concentration of plasma was increased *ca.* 4-fold in patients with eOA, *ca.* 6-fold in patients with aOA, *ca.* 3-fold in patients with eRA, *ca.* 6-fold in patients with aRA and *ca.* 4-fold in patients with non-RA, with respect to healthy controls. It was increased *ca.* 2-fold in advanced disease, comparing aOA versus eOA and aRA versus eRA. In synovial fluid of patients, MetSO free adduct concentration of plasma was increased *ca.* 5-fold in eOA, *ca.* 15-fold in aOA, *ca.* 5-fold in eRA, *ca.* 10-fold in aRA and *ca.* 4-fold in non-RA, with respect to plasma of healthy controls. NFK free adduct concentration of plasma was increased *ca.* 4-fold in patients with aOA, eRA and non-RA, and *ca.* 5 in patients with aRA, with respect to healthy controls. NFK free adduct concentration of plasma was increased *ca.* 3-fold in aOA with respect to eOA. In synovial fluid the concentration of NFK free adduct was increased *ca.* 2-fold in eOA, *ca.* 6-fold in aOA, *ca.* 16-fold in eRA, *ca.* 5-fold in aRA and *ca.* 17-fold in eRA, with respect to plasma of healthy controls . DT free adduct concentration of plasma was decreased 60% in patients with aOA, 70% in patients with eRA and 75% in patients with non-RA, with respect to healthy controls. It was decreased 80% in aOA versus eOA whereas it was increased 4-fold in aRA versus eRA. DT free adduct concentration of synovial fluid was increased *ca.* 2-fold in eOA and aOA and unchanged in other patient study groups, compared to plasma of healthy controls. Plasma 3-NT free adduct concentration was decreased 70% and 64% in plasma and synovial fluid of aOA with respect to healthy controls and unchanged in all other patient groups (Table S3).

### Glycation adducts of plasma and synovial protein

For lysine-derived adducts, FL residue content of plasma protein was increased *ca.* 2-fold in patients with eRA and non-RA, with respect to healthy controls. FL residue content of synovial fluid protein was increased *ca.* 2-fold with respect to plasma protein of patients with eOA. CML residue content of plasma protein was increased *ca.* 5-fold in patients with aOA and aRA, with respect to healthy controls. It was increased markedly in advanced disease: *ca.* 9-fold increase in aOA versus eOA and aRA versus eRA. A similar effect was found in synovial fluid protein where CML residue content was increased *ca.* 11-fold in aOA versus eOA and ca. 7-fold in aRA versus eRA. CEL residue content of plasma protein was increased *ca.* 2-fold in patients with eOA and aRA only, with respect to healthy controls. CEL residue content of synovial fluid protein was increased 2-fold with respect to plasma protein in patients with aOA. MOLD residue content of plasma protein and synovial fluid protein was unchanged in all study groups. Pentosidine residue content of plasma protein was increased *ca.* 23-fold in patients with eOA, *ca.* 9-fold in patients with aOA and *ca.* 7-fold in patients with aRA, with respect to healthy controls. Pentosidine residue content of synovial fluid protein was unchanged. In patients with aOA, pentosidine residue content of synovial fluid protein was *ca.* 7-fold higher than in plasma protein (Table S4).

For arginine-derived adducts, G-H1 residue content of plasma protein was increased *ca.* 3-fold in patients with aRA, with respect to healthy controls. G-H1 residue content of synovial fluid protein was unchanged, with respect to plasma protein healthy controls. There were *ca.* 4-fold, 2-fold and 3-fold increases in G-H1 residue content of synovial protein compared to plasma protein in patients with aOA, aRA and non-RA, respectively. MG-H1 residue content of plasma protein was decreased 64% in patients with eRA but increased 2 - 3-fold in patients with aRA; hence it increased *ca.* 7-fold in aRA versus eRA. MG-H1 residue content of synovial fluid protein was unchanged, with respect to plasma protein healthy controls. It increased *ca.* 3-fold, however, in aOA versus eOA. There were *ca.* 4-fold and 2-fold increases in MG-H1 residue content of synovial protein compared to plasma protein in patients with eRA and non-RA, respectively. 3DG-H residue content of plasma protein and synovial fluid protein was unchanged, with respect to plasma protein of healthy controls. It was increased, however, *ca.* 9-fold in plasma protein and *ca.* 4-fold in synovial protein in aOA with respect to eOA. CMA residue content of plasma protein was increased *ca.* 6-fold in aRA, *ca.* 2-fold in non-RA and was unchanged in synovial fluid protein, with respect to plasma protein of healthy controls. It was increased *ca.* 4-fold in plasma protein of aOA versus eOA (Table S5).

**Glycation free adducts of plasma and synovial fluid**

For lysine derived adducts, FL free adduct concentration in plasma and synovial fluid was little changed in the study groups except it was increased 2 – 3 fold in synovial fluid of patients with eOA. CML free adduct concentration in plasma and synovial fluid was not changed in the study groups. CEL free adduct concentration in plasma was increased *ca.* 4-fol in patients with aOA and 2-fold in patients with aRA, with respect to healthy controls. CEL free adduct concentration of synovial fluid of patients with aOA was 61% lower than in plasma. MOLD free adduct concentration in plasma was decreased 82% in patients with eOA with respect to healthy controls. MOLD free adduct concentration in synovial fluid was decreased 77% and 68% and in eOA and aOA whereas it was increased *ca.* 6-fold and 4-fold in eRA and non-RA, with respect to plasma of healthy controls. Pentosidine free adduct concentration in plasma was increased *ca.* 5-fold in patients with aRA, with respect to healthy controls. In synovial fluid it was increased *ca.* 2-fold in eOA, 3-fold in aOA and 4-fold in aRA, with respect to plasma of healthy controls. It was increased *ca.* 3-fold in aRA versus eRA in both plasma and synovial fluid (Table S6).

For arginine-derived adducts, G-H1, MG-H1 and 3DG-H free adduct concentrations in plasma were little changed in the study groups except for a 2-fold increase in MG-H1 free adduct concentration in plasma of patients with aOA and 49% decrease in 3DG-H free adduct concentration in patients with eOA. There was *ca.* 2-fold increases of G-H1 and Mg-H1 free adducts in synovial fluid of patients with eRA and a 57% decrease in 3DG-H free adduct concentration in patients with eOA, with respect to plasma of healthy controls. CMA free adduct concentration in plasma was far more responsive: it increased *ca.* 2-fold in patients with eOA, 4-fold in patients with aOA, and 3-fold in patients with eRA and non-RA, with respect to healthy controls. CMA free adduct concentration increased similarly in synovial fluid (Table S7).

**Effect of drug therapy on protein oxidation, nitration and glycation adducts in plasma and synovial fluid of patients with aRA**

Patients receiving anti-TNFα therapy, compared to those not receiving anti-TNFα therapy, had increased plasma DT free adduct (6.29 versus 4.75 nM, P<0.05) and lower synovial fluid NFK adduct free (43.2 versus 5.6 nM, P<0.05). Patients receiving treatment with non-steroidal anti-inflammatory drugs (NSAIDs), compared to those not receiving NSAIDs, had: in plasma – lower 3DG-H free adduct (181 versus 19 nM, P<0.01), MOLD free adduct (27.2 versus 0.5 nM, P<0.01), 3-NT free adduct (3.7 versus 0.7 nM, P<0.01), 3DG-H protein adduct (0.245 versus 0.014 mmol/mol arg, P<0.05) and MetSO protein adduct (30 versus 14 mmol/mol met, P<0.05) but higher DT free adduct (5.1 versus 7.1 nM) and CEL protein adduct (0.030 versus 0.099 mmol/mol arg, P<0.05); and in synovial fluid – lower G-H1 free adduct (60 versus 19 nM, P<0.01) but higher CEL protein adduct (0.028 versus 0.094 mmol/mol arg, P<0.05). Patients receiving treatment with prednisolone, compared to those not receiving prednisolone, had: in plasma – higher CML free adduct (230 versus 99 nM, P<0.05), 3DG-H and MetSO protein adduct (0.283 versus 0.016 mmol/mol arg and 47 versus 16 mmol/mol met, respectively, P<0.05); and in synovial fluid – lower CEL free adduct (69 versus 453 nM, P<0.05) but high 3DG-H free adduct (253 versus 26 nM, P<0.05). Treatment with methotrexate was associated with increased MG-H1 free adduct in plasma and synovial fluid (plasma: 307 versus 665 nM, P<0.05; synovial fluid, 308 versus 829 nM, P<0.01) and decreased synovial fluid 3-NT residue content (0.0053 versus 0.0022 mmol/mol tyr, P<0.05).

Table S1 Mass spectrometric multiple reaction monitoring detection of protein oxidation, nitration and glycation adducts.

| Analyte group | Analyte | Rt (min) | Parent ion Da) | Fragment  ion (Da) | CE  (eV) | Neutral fragment loss(es) | Isotopic standard (amount) |
| --- | --- | --- | --- | --- | --- | --- | --- |
| Oxidation adducts | MetSO | 8.7 | 166.1 | 102.2 | 14 | CH_3_-SOH | [^2^H_3_]MetSO (5 pmol) |
|  | DT | 19.9 | 361.2 | 315.3 | 15 | H_2_CO_2_ | [^2^H_6_]DT (1 pmol) |
|  | NFK | 21.5 | 235.8 | 191.2 | 18 | H_2_CO_2_ | [^15^N_2_]NFK (1 pmol) |
| Nitration adduct | 3-NT | 23.2 | 227.1 | 181.2 | 13 | H_2_CO_2_ | [^2^H_3_]3-NT (1 pmol) |
| Early glycation | FL | 28.5 | 291.0 | 84.3 | 31 | H_2_CO_2_, fructosylamine | [^2^H_4_]FL (10 pmol) |
| AGE | CML | 28.5 | 204.9 | 130.1 | 12 | NH_2_CH_2_CO_2_H | [^13^C_6_]CML (1 pmol) |
|  | CEL | 28.8 | 219.2 | 130.1 | 13 | NH_2_CH(CH_3_)CO_2_H | [^13^C_6_]CEL (1 pmol) |
|  | CMA | 12.1 | 233.0 | 70.1 | 27 | H_2_CO_2_, NH_2_C(=NH)NHCH_2_CO_2_H | [^13^C_2_]CMA (1 pmol) |
|  | G-H1 | 12.4 | 215.0 | 100.2 | 14 | NH_2_CH(CO_2_H)CH_2_CH=CH_2_ | [^15^N_2_]G-H1 (5 pmol) |
|  | MG-H1† | 11.6 & 12.5 | 229.2 | 114.3 | 14 | NH_2_CH(CO_2_H)CH_2_CH=CH_2_ | [^15^N_2_]MG-H1 (5 pmol) |
|  | 3DG-H† | 11.2, 12.6 & 13.5 | 319.1 | 114.8 | 20 | NH_2_CH(CO_2_H)CH_2_CH=CH_2_ | [^15^N_2_]3DG-H (5 pmol) |
|  | Pentosidine‡ | 21.1 | 379.3 | 250.4 | 22 | NH_2_CH(CO_2_H)CH_2_CH_2_CH=CH_2_ | [^13^C_6_]pentosidine |

Table S1 Mass spectrometric multiple reaction monitoring detection of protein oxidation, nitration and glycation adducts (cont’d).

| Analyte group | Analyte | Rt (min) | Parent ion Da) | Fragment  ion (Da) | CE  (eV) | Neutral fragment loss(es) | Isotopic standard |
| --- | --- | --- | --- | --- | --- | --- | --- |
| AGE | MOLD | 14.0 | 341.2 | 212.3 | 21 | NH_2_CH(CO_2_H)CH_2_CH_2_CH=CH_2_ | [^2^H_8_]MOLD (1 pmol) |
| Amino acids | Hyp | 5.6 | 132.0 | 86.1 | 12 | H_2_CO_2_ | 4,5-[^13^C_2_]Hyp (5 pmol) |
|  | Arg | 29.2 | 175.2 | 70.3 | 15 | H_2_CO_2_, NH_2_C(=NH)NH_2_ | [^15^N_2_]arg (1 nmol) |
|  | Lys | 5.5 | 147.1 | 84.3 | 15 | H_2_CO_2_, NH_3_ | [^13^C_6_]lys (1 nmol) |
|  | Met | 29.5 | 150.0 | 104.2 | 11 | H_2_CO_2_ | [^2^H_3_]met (1 nmol) |
|  | Tyr | 18.3 | 182.1 | 136.2 | 13 | H_2_CO_2_ | [^2^H_4_]tyr (1 nmol) |
|  | Trp | 23.5 | 205.0 | 159.1 | 15 | H_2_CO_2_ | [^15^N_2_]trp (0.2 nmol) |
|  | Val | 8.6 | 117.8 | 72.0 | 19 | H_2_CO_2_ | [^2^H_8_]val (1 nmol) |

†For hydroimidazolones, R_t_ values for the 2 epimers if MG-H1 are given and of the 3 structural isomers of 3DG-H, 3DG-H1, 3DG-H2 and 3DG-H3 are all detected. **‡** Pentosidine is detected to higher sensitivity by in-line fluorimetry, excitation wavelength 320 nm, emission wavelength 365 nm. Data from: Thornalley PJ, Rabbani N. Detection of oxidized and glycated proteins in clinical samples using mass spectrometry - A user's perspective. Biochim Biophys Acta. 2014;1840(2):818-29; and Rabbani N, Shaheen F, Anwar A, Masania J, Thornalley PJ. Assay of methylglyoxal-derived protein and nucleotide AGEs. Biochem Soc Trans 2014;42(2):511-7.

Table S2 Oxidation and nitration adduct residues of protein in plasma and synovial fluid (training set for machine learning).

| Compartment | Group | MetSO (mmol/mol met) | NFK (mmol/mol trp) | DT (mmol/mol tyr) | 3-NT (mmol/mol tyr) |
| --- | --- | --- | --- | --- | --- |
| Plasma | Control (n = 16) | 7.6 (5.3 – 10.1) | 0.205 (0.076 – 0.593) | 0.0038 (0.0024 - 0.0082) | 0.0054 (0.0030 - 0.0086) |
|  | eOA (n = 16) | 15.3 (11.8 – 18.5)*** | 0.107 (0.074 – 0.148) | 0.0069 (0.0028 - 0.0137) | 0.0008 (0.0002 - 0.0019)** |
|  | aOA (n = 17) | 21.3 (13.7 - 28.6)*** | 0.551 (0.442 – 0.763)^OOO^ | 0.2083 (0.1038 – 0.2553)***^,OOO^ | 0.0077 (0.0029 – 0.0089)^OOO^ |
|  | eRA (n = 10) | 15.7 (14.3 – 17.7)*** | 0.294 (0.167 – 0.360) | 0.0012 (0.0004 – 0.0021)* | 0.0009 (0.0007 - 0.0011)** |
|  | aRA (n = 22) | 17.4 (13.1 - 41.4)*** | 0.390 (0.199 - 0.720) | 0.0171 (0.0081 - 0.0570)**’ ^OOO^ | 0.0035 (0.0017 - 0.0054) |
|  | Non-RA (n = 10) | 18.2 (15.3 – 19.2)*** | 0.097 (0.068 - 0.128) | 0.0010 (0.0007 - 0.0018)** | 0.0011 (0.0008 – 0.0016)* |
|  | Significance | P<0.001 | P<0.001 | P<0.001 | P<0.001 |
| Synovial fluid | eOA (n = 16) | 18.3 (14.9 – 24.4)*** | 0.125 (0.048 - 0.405) | 0.0040 (0.0011 - 0.0101) | 0.0009 (0.0006 - 0.0016)*** |
|  | aOA (n = 17) | 22.9 (17.5 – 26.8)*** | 0.440 (0.258 – 0.915)^OO^ | 0.3522 (0.2070 – 0.5433)***,^OOO^ | 0.0220 (0.0050 - 0.0300) *,^OOO^,† |
|  | eRA (n = 10) | 3.6 (3.3 – 4.8)*,††† | 0.026 (0.015 – 0.055) ***,†† | 0.0013 (0.0009 - 0.0018)* | 0.0046 (0.0038 – 0.0059)†† |
|  | aRA (n = 22) | 10.4 (8.2 - 32.4)^OOO^ | 0.274 (0.153 - 0.455) | 0.0374 (0.0112 - 0.1258)*^,OOO^ | 0.0032 (0.0018 - 0.0067) |
|  | Non-RA (n = 10) | 5.1 (3.6 - 9.4) ††† | 0.034 (0.012 – 0.047)*** | 0.0007 (0.0005 – 0.0009)*** | 0.0037 (0.0027 – 0.0066) |
|  | Significance | P<0.001 | P<0.001 | P<0.001 | P<0.001 |

Data are median (lower – upper quartile). Significance: P-values for 5-group plasma and 4-group synovial fluid comparisons are given above; *Kruskal-Wallis.* For binary comparisons: *, ** and ***, P<0.05, P<0.01 and P<0.001 with respect to plasma levels of healthy controls; oo and ooo, P<0.01 and P<0.001 for aRA and aOA with respect to eRA and eOA, respectively; and †, †† and †††, P<0.05, P<0.01 and P<0.001 for synovial fluid with respect to plasma of the same study group; *Mann-Whitney U.* A Bonferroni correction of 13 was applied.

Table S3 Oxidation and nitration free adducts of plasma and synovial fluid (training set for machine learning).

| Compartment | Study group | MetSO (nM) | NFK (nM) | Dityrosine (nM) | 3-NT (nM) |
| --- | --- | --- | --- | --- | --- |
| Plasma | Control (n = 16) | 22.4 (15.4 – 37.5) | 3.46 (1.84 – 4.85) | 4.56 (4.04 – 7.78) | 1.85 (1.02 – 2.76) |
|  | eOA (n = 16) | 84.1 (74.8 – 133.6)*** | 4.93 (2.81 – 8.97) | 8.95 (4.44 – 9.89) | 1.12 (1.05 – 1.19) |
|  | aOA (n = 17) | 140.4 (100.0 – 300.2)***^,OO^ | 14.34 (10.58 – 28.75)***^,OOO^ | 1.82 (1.29 – 3.24),***^,OOO^ | 0.55 (0.43 – 0.89)*** ^,^ ^OOO^ |
|  | eRA (n = 10) | 67.0 (54.8 – 81.9)*** | 13.97 (10.74 – 23.89)** | 1.36 (1.15 – 1.63)*** | 1.26 (1.14 – 1.43) |
|  | aRA (n = 22) | 124.0 (99.9 – 202.4)***^,OOO^ | 16.24 (10.21 – 20.45)*** | 5.55 (4.49 – 6.64) ^OOO^ | 1.79 (0.78 – 4.15) |
|  | Non-RA (n = 10) | 94.5 (61.5 – 118.6)*** | 13.62 (8.52 – 15.11)** | 1.15 (0.87 – 1.36)*** | 1.49 (1.26 – 2.23) |
|  | Significance | P<0.001 | P<0.001 | P<0.001 | P>0.05 |
| Synovial fluid | eOA (n = 16) | 120 (69 – 182)*** | 6.72 (4.96 – 8.69)*** | 7.95 (5.79 – 9.85)** | 0.97 (0.83 – 1.11) |
|  | aOA (n = 17) | 335 (233 – 469)***, ^OO^ | 19.6 (12.2 – 28.1) ***,^OO^ | 9.20 (7.80 – 12.40)***,† | 0.66 (0.50 – 0.84)*** |
|  | eRA (n = 10) | 120 (80 – 157) *** | 55.1 (42.6 – 62.4)*** | 1.25 (0.92 – 1.59) | 1.46 (1.29 – 1.59) |
|  | aRA (n = 22) | 225 (134 – 283)*** | 18.1 (14.1 – 41.7) *** | 6.03 (5.10 – 7.63) ^OOO^ | 2.25 (0.63 – 5.25) |
|  | Non-RA (n = 10) | 88 (71 – 99) *** | 60.0 (42.3 – 81.3)*** | 1.04 (0.90 – 1.39) | 1.52 (1.37 – 1.93) |
|  | Significance | P<0.001 | P<0.001 | P<0.001 | P<0.001 |

Data are median (lower – upper quartile). Significance: P-values for 5-group plasma and 4-group synovial fluid comparisons are given above; *Kruskal-Wallis.* For binary comparisons: ** and ***, P<0.01 and P<0.001 with respect to plasma levels of healthy controls; oo and ooo, P<0.01 and P<0.001 for aRA and aOA with respect to eRA and eOA, respectively;†, P<0.05 for synovial fluid with respect to plasma of the same study group; *Mann-Whitney U.* A Bonferroni correction of 13 was applied.

Table S4 Lysine-derived glycation adduct residues in plasma and synovial protein (training set for machine learning).

| Compartment | Group | FL | CML | CEL | MOLD | Pentosidine |
| --- | --- | --- | --- | --- | --- | --- |
| Plasma | Control (n = 16) | 4.63 (3.51 – 6.01) | 0.075 (0.043 – 0.135) | 0.019 (0.016 – 0.025) | 0.0014 (0.0006 - 0.0103) | 0.0005 (0.0002 – 0.0007) |
|  | eOA (n = 16) | 4.72 (4.56 – 4.96) | 0.044 (0.033 - 0.051) | 0.046 (0.014 - 0.064) * | 0.0030 (0.0017 – 0.0033) | 0.0117 (0.0009 – 0.0209)*** |
|  | aOA (n = 17) | 5.12 (4.63 – 5.97) | 0.401 (0.165 - 0.674)***^,OOO^ | 0.023 (0.016 – 0.030) | 0.0047 (0.0011 - 0.0103) | 0.0047 (0.0011 - 0.0103)** |
|  | eRA (n = 10) | 7.59 (6.99 – 8.10)*** | 0.045 (0.029 – 0.048) | 0.033 (0.023 – 0.051) | 0.0033 (0.0025 – 0.0050) | 0.0024 (0.0006 - 0.0051) |
|  | aRA (n = 22) | 5.73 (5.02 – 6.35)^OOO^ | 0.389 (0.146 - 0.474)***^,OOO^ | 0.035 (0.020 - 0.103) * | 0.0037 (0.0020 - 0.0080) | 0.0037 (0.0027 - 0.0094) *** |
|  | Non-RA (n = 10) | 8.13 (7.18 -8.68)*** | 0.033 (0.023 – 0.037) | 0.028 (0.013 – 0.036) | 0.0035 (0.0029 – 0.0051) | 0.0014 (0.0008 – 0.0027) |
|  | Significance | P<0.001 | P<0.001 | P>0.05 | P>0.05 | P<0.001 |
| Synovial fluid | eOA (n = 16) | 8.82 (7.16 - 10.58) † | 0.050 (0.042 - 0.081) | 0.042 (0.013 - 0.075) | 0.0028 (0.0021 – 0.0060) | 0.0110 (0.0033 – 0.0129) |
|  | aOA (n = 17) | 6.00 (5.13 – 6.75)^OOO^ | 0.537 (0.301- 0.650)^OOO^ | 0.056 (0.029 - 0.103) † | 0.0130 (0.080 – 0.017)^OOO^ | 0.0341 (0.0117 - 0.0633) ††† |
|  | eRA (n = 10) | 6.19 (5.27 – 6.75) | 0.050 (0.042 – 0.060) | 0.024 (0.021 – 0.033) | 0.0046 (0.0018 – 0.0063) | 0.0032 (0.0019 – 0.0041) |
|  | aRA (n = 22) | 6.53 (5.29 – 7.82) | 0.349 (0.156 - 0.479) ^OOO^ | 0.045 (0.022 - 0.082) | 0.0054 (0.0027 – 0.0081) | 0.0069 (0.0040 - 0.0095) |
|  | Non-RA (n = 10) | 6.41 (5.29 – 7.04) | 0.049 (0.034 – 0.071) | 0.026(0.021 – 0.033) | 0.0044 (0.0023 – 0.0062) | 0.0023 (0.0017 – 0.0030) |
|  | Significance | P<0.01 | P<0.001 | P>0.05 | P<0.001 | P<0.001 |

Data are median (lower – upper quartile); mmol/mol lys. Significance: P-values for 5-group plasma and 4-group synovial fluid comparisons are given above; *Kruskal-Wallis.*For binary comparisons: *, ** and ***, P<0.05, P<0.01 and P<0.001 for plasma levels with respect to Control; ooo, P P<0.001 for aRA and aOA with respect to eRA and eOA, respectively;† and †††, P<0.05and P<0.001 for synovial fluid with respect to plasma of the same study group; *Mann-Whitney U.* A Bonferroni correction of 13 was applied.

**Table S5** Arginine-derived glycation adduct residues in plasma and synovial protein (training set for machine learning).

| Compartment | Study group | G-H1 | MG-H1 | 3DG-H | CMA |
| --- | --- | --- | --- | --- | --- |
| Plasma | Control (n = 16) | 0.023 (0.014 – 0.046) | 0.288 (0.126 – 0.374) | 0.085 (0.022 – 0.309) | 0.024 ± 0.007 |
|  | eOA (n = 16) | 0.031 (0.024 – 0.036) | 0.257 (0.242 – 0.339) | 0.022 (0.016 - 0.046) | 0.015 (0.008 - 0.026) |
|  | aOA (n = 17) | 0.070 (0.008 - 0.139) | 0.574 (0.174 - 1.175) | 0.207 (0.043 - 0.524) ^OO^ | 0.033 (0.019 – 0.051) |
|  | eRA (n = 10) | 0.021 (0.015 - 0.040) | 0.104 (0.091 – 0.128)* | 0.031 (0.023 – 0.035) | 0.034 (0.025 – 0.044) |
|  | aRA (n = 22) | 0.081 (0.045 - 0.182)***^,OO^ | 0.720 (0.386 – 1.208)*^,OOO^ | 0.096 (0.013 - 0.283) | 0.144 (0.105 – 0.268)***^,OOO^ |
|  | Non-RA (n = 10) | 0.028 (0.016 - 0.047) | 0.138 (0.129 – 0.160) | 0.021 (0.018 – 0.024) | 0.045 (0.034 – 0.061)* |
|  | Significance | P<0.001 | P<0.001 | P<0.01 | P<0.001 |
| Synovial fluid | eOA (n = 16) | 0.046 (0.035 – 0.061) | 0.353 (0.276 - 0.421) | 0.059 (0.041 – 0.067) | 0.050 (0.033 – 0.059) |
|  | aOA (n = 17) | 0.253 (0.204 – 0.408)^OOO,^† | 0.900 (0.449 - 1.300) ^OOO^ | 0.200 (0.157 – 0.260)^OOO^ | 0.078 (0.038 – 0.104) |
|  | eRA (n = 10) | 0.090 (0.034 - 0.128) | 0.429 (0.262 – 0.563)† | 0.036 (0.029 - 0.057) | 0.043 (0.034 - 0.055) |
|  | aRA (n = 22) | 0.195 (0.078 - 0.456)† | 0.653 (0.332 - 1.119) | 0.168 (0.018 - 0.415) | 0.088 (0.036 - 0.189) |
|  | Non-RA (n = 10) | 0.083 (0.066 - 0.131)† | 0.288( 0.252 – 0.371)†† | 0.039 (0.030 – 0.043) | 0.045 (0.031 – 0.049) |
|  | Significance | P<0.001 | P<0.01 | P<0.01 | P>0.05 |

Data are median (lower – upper quartile); mmol/mol arg. Significance: P-values for 5-group plasma and 4-group synovial fluid comparisons are given above; *Kruskal-Wallis.* For binary comparisons: * and ***, P<0.05 and P<0.001 for plasma levels with respect to Control; oo and ooo, P<0.01 and P<0.001 for aRA and aOA for eRA and eOA, respectively; and † and ††, P<0.05 and P<0.01 for synovial fluid with respect to plasma of the same study group; *Mann-Whitney U.* A Bonferroni correction of 13 was applied.

Table S6 Lysine-derived glycation free adducts in plasma and synovial fluid (training set for machine learning).

| Compartment | Study group | FL (nM) | CML (nM) | CEL (nM) | MOLD (nM) | Pentosidine (nM) |
| --- | --- | --- | --- | --- | --- | --- |
| Plasma | Control (n = 16) | 72.4 (64.4 – 104.7) | 89.6 (18.6 – 196.1) | 89.6 (33.5 – 135.6) | 1.54 (1.04 – 2.82) | 0.67 (0.37 – 1.24) |
|  | eOA (n = 16) | 84.1 (74.8 – 133.6) | 124.4 (87.2 – 164.7) | 88.8 (79.9 – 122.3) | 0.27 (0.17 – 0.35)*** | 1.01 (0.67 – 1.51) |
|  | aOA (n = 17) | 99.1 (53.6 – 172.5) | 127.4 (191.8 – 215.7) | 353.7 (134.7 – 715.2)***^, OO^ | 0.60 (0.21– 1.26) | 2.57 (1.51 – 3.59) |
|  | eRA (n = 10) | 71.6 (61.5 – 79.7) | 133.5 (72.7 – 165.0) | 89.7 (67.6 – 104.6) | 2.16 (1.82 – 2.70) | 1.02 (0.83 – 1.36) |
|  | aRA (n = 22) | 88.8 (69.7 – 164.0) | 121 (90 – 224) | 190.6 (115.4 – 400.5)* | 7.01 (0.47 – 35.13) | 3.23 (2.45 – 3.81)***^,OO^ |
|  | Non-RA (n = 10) | 71.9 (47.2 – 83.6) | 118.5 (86.9 – 176.5) | 94.4 (72.1 – 113.7) | 2.09 (1.71 – 2.63) | 0.88 (0.64 – 1.13) |
|  | Significance | P>0.05 | P>0.05 | P<0.001 | P<0.001 | P<0.001 |
| Synovial fluid | eOA (n = 16) | 185.4 (108.3 – 202.6)* | 81.9 (51.7 – 132.7) | 99.5 (79.1 – 123.1) | 0.36 (0.24 – 0.57) *** | 1.54 (1.36 – 1.71)*** |
|  | aOA (n = 17) | 59.4 (41.6 – 72.8) ^OO,^ † | 106.3 (71.7 – 140.5) | 138.4 (97.3 – 332.5) †† | 0.50 (0.01 – 0.80) *** | 1.89 (1.51 – 3.38)*** |
|  | eRA (n = 10) | 92.5 (76.9 – 120.9) | 153 (99 – 206) † | 91.9 (73.9 – 111.2) | 8.55 (7.16 – 8.78)*** | 0.80 (0.71 – 1.07) |
|  | aRA (n = 22) | 101.8 (59.8 – 232.6) | 244 (138 – 325) | 153.4 (75.2 – 463.5) | 1.29 (0.57 – 1.74) ^OO^ | 2.47 (2.19 – 5.84) ^OOO^,*** |
|  | Non-RA (n = 10) | 68.4 (61.5 – 88.4) | 118 (94 – 162) | 82.6 (70.5 – 91.1) | 6.41 (3.38 – 11.78)*** | 0.85 (0.69 – 1.38) |
|  | Significance | P<0.05 | P<0.001 | P>0.05 | P<0.001 | P<0.001 |

Data are median (lower – upper quartile). Significance: P-values for 5-group plasma and 4-group synovial fluid comparisons are given above; *Kruskal-Wallis.* For binary comparisons: * and ***, P<0.05 and P<0.001 with respect to plasma levels of healthy controls; oo and ooo, P<0.01 and P<0.001 for aRA and aOA with respect to eRA and eOA, respectively;† and ††, P<0.05 and P<0.01 for synovial fluid with respect to plasma of the same study group; *Mann-Whitney U*. A Bonferroni correction of 13 was applied.

Table S7 Arginine-derived glycation free adducts in plasma and synovial fluid (training set for machine learning).

| Compartment | Group | G-H1 (nM) | MG-H1 (nM) | 3DG-H (nM) | CMA (nM) |
| --- | --- | --- | --- | --- | --- |
| Plasma | Control (n = 16) | 49.1 (36.8 – 71.1) | 260 (131 – 447) | 66.7 (41.5 – 112.0) | 5.8 (3.4 – 6.7) |
|  | eOA (n = 16) | 55.3 (41.7 – 76.6) | 278 (226 – 496) | 34.2 (25.6 – 47.6)* | 6.17 (4.68 – 9.76) |
|  | aOA (n = 17) | 114.5 (34.8 – 256.1) | 511 (306 – 766)* | 69.5 (21.1 – 99.6) | 14.2 (12.3 – 20.1)***^,OOO^ |
|  | eRA (n = 10) | 70.4 (60.8 – 103.7) | 551 (303 – 1160) | 57.3 (43.7 – 63.3) | 21.1 (14.4 – 27.8)*** |
|  | aRA (n = 22) | 45.8 (35.8 – 94.6) | 597 (314 – 742) | 58.0 (18.5 – 193.4) | 16.1 (10.7 – 22.9)*** |
|  | Non-RA (n = 10) | 78.4 (56.1 – 90.4) | 438 (383 – 906) | 60.8 (47.6 – 76.6) | 16.9 (14.9 – 25.3)*** |
|  | Significance | P>0.05 | P<0.05 | P>0.05 | P<0.001 |
| Synovial fluid | eOA (n = 16) | 56.3 (48.6 – 72.9) | 223 (198 – 325) | 34.2 (30.9 – 50.3) | 7.2 (6.2 – 10.1) |
|  | aOA (n = 17) | 83.9 (39.6 – 154.6) | 360 (275 – 656) | 29.0 (23.0 – 46.3)*** | 12.9 (7.3 – 17.6)*** |
|  | eRA (n = 10) | 101.0 (84.2 – 126.3)*** | 615 (367 – 1343)* | 50.5 (41.0 – 72.7) | 17.5 (11.7 – 26.6)*** |
|  | aRA (n = 22) | 44.0 (19.7 – 77.8) | 562 (289 – 838) | 107.3 (23.9 – 251.9) | 13.4 (9.9 – 19.5)*** |
|  | Non-RA (n = 10) | 76.7 (67.1 – 117.4) | 462 (318 – 672) | 39.9 (30.7 – 57.6) | 16.0 (13.3 – 22.5)*** |
|  | Significance | P>0.05 | P<0.05 | P>0.05 | P<0.001 |

Data are median (lower – upper quartile). Significance: P-values for 5-group plasma and 4-group synovial fluid comparisons are given above; *Kruskal-Wallis.* For binary comparisons: * and ***, P<0.05 and P<0.001 with respect to plasma levels of healthy controls; ooo, P<0.001 for aRA and aOA with respect to eRA and eOA, respectively; *Mann-Whitney U*. A Bonferroni correction of 13 was applied.

Table S8 Oxidation and nitration free adducts of plasma (test set for algorithm validation).

| Compartment | Study group | MetSO (nM) | NFK (nM) | Dityrosine (nM) | 3-NT (nM) |
| --- | --- | --- | --- | --- | --- |
| Plasma | Control (n = 37) | 99 (81 – 121) | 19.1 (13.9 – 30.6) | 2.17 (1.43 – 2.61) | 0.388 (0.184 – 0.609) |
|  | eOA (n = 30) | 179 (61 – 101)** | 13.8 (11.0 – 20.8) * | 1.77 (1.26 – 2.50) | 0.469 (0.138 – 0.612) |
|  | eRA (n = 35) | 155 (105 – 222)*** | 6.4 (4.7 – 10.0)*** | 1.35 (0.85 – 1.81)* | 0.895 (0.692 – 1.021)*** |
|  | Non-RA (n = 32) | 145 (123– 230)*** | 7.4 (4.8 – 11.4)*** | 1.32 (1.15 – 1.63)*** | 0.883 (0.734 – 1.009)*** |
|  | Significance | P<0.001 | P<0.001 | P>0.05 | P<0.001 |

Data are median (lower – upper quartile). Significance: P-values for 4 study groups are given above; *Kruskal-Wallis.* For binary comparisons: *, ** and ***, P<0.05, P<0.01 and P<0.001 with respect to plasma levels of healthy controls; *Mann-Whitney U*. A Bonferroni correction of 13 was applied.

Table S9 Lysine-derived glycation free adducts in plasma (test set for algorithm validation).

| Compartment | Study group | FL (nM) | CML (nM) | CEL (nM) | Pentosidine (nM) |
| --- | --- | --- | --- | --- | --- |
| Plasma | Control (n = 37) | 549 (462 – 778) | 216 (165 – 282) | 39 (20 – 73) | 0.218 (0.148 – 0.284) |
|  | eOA (n = 30) | 419 (61 – 646)** | 239 (145 – 306) | 72 (38 – 233)** | 0.301 (0.233 – 0.599)** |
|  | eRA (n = 35) | 457 (320 – 830) | 114 (89 – 156)*** | 255 (195 – 384)*** | 0.810 (0.570 – 0.940)*** |
|  | Non-RA (n = 32) | 417 (307 – 664)* | 107 (80 – 150)*** | 330 (234 – 425)*** | 0.810 (0.563 – 1.048)*** |
|  | Significance | P>0.05 | P<0.001 | P<0.001 | P<0.001 |

Data are median (lower – upper quartile). Significance: P-values for 4 study groups are given above; *Kruskal-Wallis.* For binary comparisons: *, ** and ***, P<0.05, P<0.01 and P<0.001 with respect to plasma levels of healthy controls; *Mann-Whitney U*. A Bonferroni correction of 13 was applied.

Table S10 Arginine-derived glycation free adducts in plasma (test set for algorithm validation).

| Compartment | Study group | G-H1 (nM) | MG-H1 (nM) | 3DG-H (nM) | CMA (nM) |
| --- | --- | --- | --- | --- | --- |
| Plasma | Control (n = 37) | 55.4 (28.1 – 73.7) | 430 (284 – 817) | 54.0 (26.0 – 92.0) | 22.4 (15.4 – 30.8) |
|  | eOA (n = 30) | 40.4 (17.1 – 71.3) | 401 (272 – 527) | 77.0 (49.8 – 118.8)* | 23.7 (10.6 – 34.5) |
|  | eRA (n = 35) | 32.3 (22.7 – 44.4)** | 321 (161 – 553)* | 41.0 (29.0 – 75.0) | 7.4 (4.2 – 13.9)*** |
|  | Non-RA (n = 32) | 29.4 (23.3 – 41.1)** | 240 (150 – 415)*** | 38.5 (23.5 – 58.0) | 5.0 (2.6 – 7.0)*** |
|  | Significance | P>0.05 | P<0.05 | P>0.05 | P<0.001 |

Data are median (lower – upper quartile). Significance: P-values for 4 study groups are given above; *Kruskal-Wallis.* For binary comparisons: *, ** and ***, P<0.05, P<0.01 and P<0.001 with respect to plasma levels of healthy controls; *Mann-Whitney U*. A Bonferroni correction of 13 was applied.


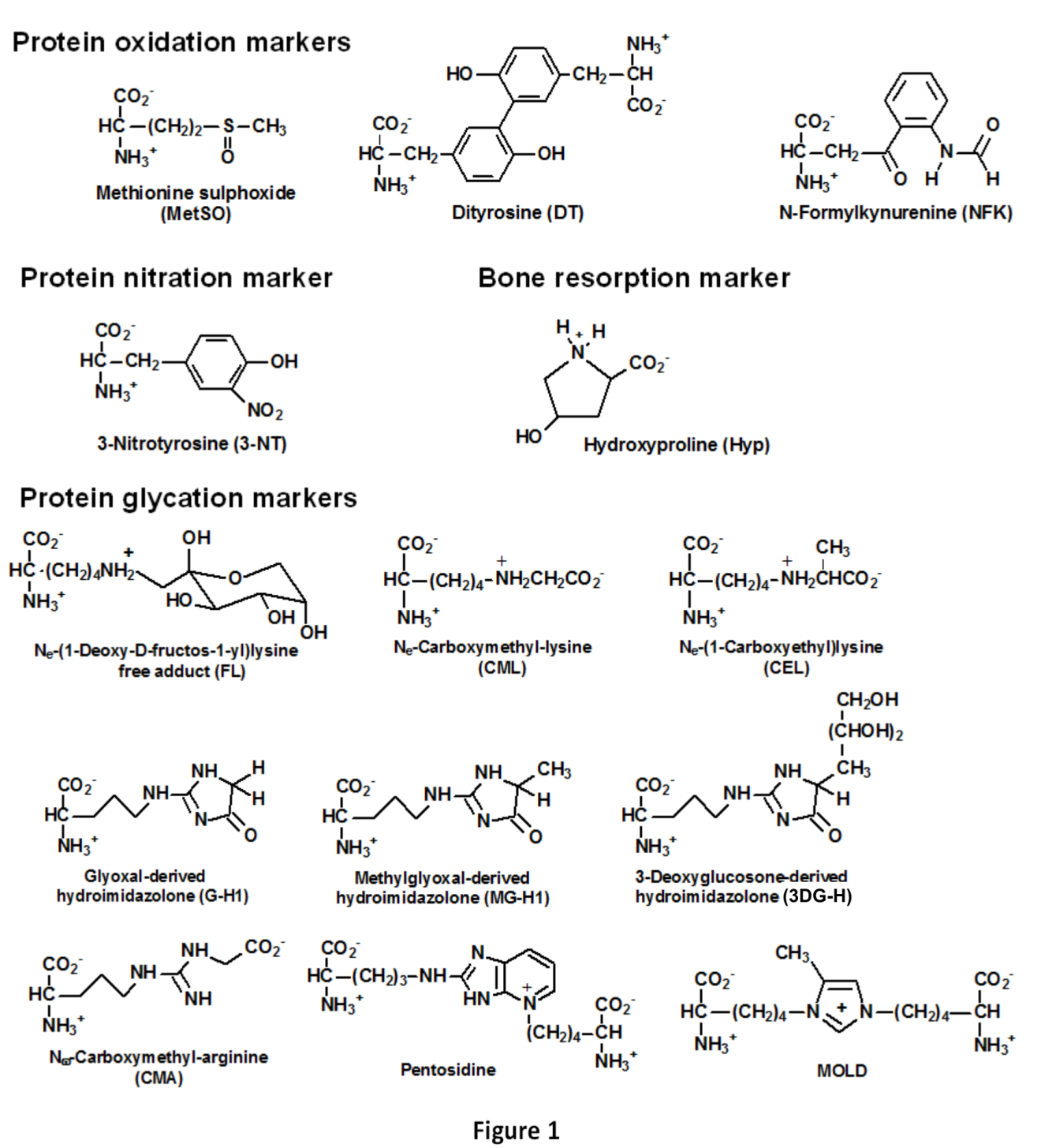


**Fig. S1** Oxidised, nitrated and glycated amino acids markers and the bone resportion marker, hydroxyproline. For the corresponding glycation, oxidation and nitration adducts of proteins the NH_3_^+^- and -CO_2_^-^ termini are part of peptide backbone of the protein as -NH- and –CO- residues, respectively.
